# Immediate, Short-Term, Intermediate, and Long-Term Clinical Outcomes of True Bifurcation Stenting

**DOI:** 10.7759/cureus.67251

**Published:** 2024-08-19

**Authors:** Mamoon Qadir, Anwar Ali, Fahad Khalid, Bakht Umar Khan, Iqbal Saifullah Khan, Amna Akbar, Sarosh Khan Jadoon, Sabahat Tasneem

**Affiliations:** 1 Interventional Cardiology, Fellowship of the Royal College of Physicians (FRCP, UK) Kulsum International Hospital, Polyclinic Hospital Islamabad, Islamabad, PAK; 2 CT Angiography, Kulsum International Hospital, Islamabad, PAK; 3 Cardiology, Federal Government Polyclinic Hospital Islamabad, Islamabad, PAK; 4 Interventional Cardiology, Armed Forces Institute of Cardiology and National Institute of Heart Diseases, Rawalpindi, PAK; 5 Interventional Cardiology, Chairman Kulsum International Hospital, Islamabad, PAK; 6 Medical Emergency and Accident, District Headquarter Hospital, Jhelum Valley, Muzaffarabad, PAK; 7 General Surgery, Combined Military Hospital, Muzaffarabad, PAK; 8 Public Health, Health Services Academy, Islamabad, PAK

**Keywords:** drug-eluting stent, acute coronary syndrome (acs), major adverse cardiac effect (mace), major adverse cardiac events, bifurcation lesion

## Abstract

Introduction: Coronary artery bifurcation lesion is an epicardial stenosis that, when compared to non-bifurcation lesions, poses a greater risk of adverse events and can compromise prognosis. This study aims to investigate the clinical efficacy of different stenting techniques, particularly in terms of their immediate, short-term, intermediate, and long-term outcomes in patients with true bifurcation lesions.

Methodology: This retrospective observational cohort study was conducted in a tertiary cardiac hospital in Islamabad, from February 1, 2015, to February 28, 2021. A total of 172 patients who met the inclusion criteria and underwent percutaneous coronary intervention were selected using a consecutive sampling technique. Follow-up was maintained for three years to assess procedural outcomes.

Results: Of the 172 participants, the majority were males (69%) and only 4% were above 75 years of age. A significant relation between major adverse cardiac events (MACEs) with acute coronary syndrome (ACS) and previous percutaneous coronary intervention (PCI) (p < 0.000) was observed. Procedural success was good in all patients using the drug-eluting stent. The MAC rate was 6.9% and the final kissing balloon inflation, stenting technique, and bifurcation involvement were significantly associated with MACE occurrence (p < 0.01), and mortality was reported in two patients (1.16%). MACEs were associated with mortality; previous PCI and hypertension increased the risk of mortality.

Conclusion: The two-stent strategy can be used with good long-term outcomes and low complication rates.

## Introduction

Coronary bifurcation lesion is an epicardial stenosis that must be percutaneously vascularized [1]. A coronary artery bifurcation lesion is characterized by constriction of the coronary arteries adjoining or/and engaging the origin of a significant side branch (SB). A major SB is one whose loss has implications for a specific patient's symptoms, left ventricular function, collateralizing vascular function, viability of the supplied myocardium, and the site of ischemia [2]. Based on angiographic results, numerous classifications of bifurcations have been proposed. The Medina classification, which shows the site of considerable stenosis (i.e., stenosis > 50%) in bifurcation, is one of the most popular and straightforward. Despite its limitations, it has been stated that any BIF stenting technique would benefit from considering aspects such as calcification, bifurcation angles, lesion length, and practical significance of the lesions. Fractional flow reserve (FFR) or intravascular ultrasound imaging can be used to further define Medina categorization. The risk of major adverse cardiac events (MACE) is significantly associated with bifurcation lesions as compared to non-bifurcation lesions [3], and catheter-based treatment can be technically challenging. As a result, coronary bifurcation lesions are crucial and account for approximately 15-20% of all PCIs [4-7].

The clinical results of bifurcation stenting through percutaneous revascularization are significantly influenced by the anatomy of the bifurcation, including the main branch (MB) size relative to the SB, angle of the bifurcation, significance of the SB, and extent of the disease in the SB. To properly analyze trials, the anatomical characteristics of bifurcation lesions should also be considered [7]. According to recent investigations, anatomically acceptable bifurcation lesions should be treated using provisional stenting. The clinical use of cutting-edge provisional stenting methods improves the safety of SB. The double kissing crush (DK-Crush) technique is preferred when a two-stent approach is necessary, particularly in left major bifurcations. However, intravascular imaging is of utmost significance for bifurcation PCI procedure success [8]. The results of PCI for bifurcation lesions have significantly improved over the recent decade as a result of advancements in device technology, operative procedures, and prophylactic antithrombotic medications [9,10]. Stenting of the coronary bifurcation is complicated and poses a high risk of stent thrombosis and restenosis, despite the development of procedures such as the use of drug-eluting stents (DES) [11]. The use of a single stent or the provisional method has been proven preferable to the use of two stents (elective) [12] and to produce better results in cases of peri-procedural myocardial infarction (MI) [13]. For bifurcation stenting, this is the procedure of choice.

Interventional cardiologists frequently encounter coronary artery bifurcation lesions and there is great risk for MACE. Therefore, the present three-year follow-up study offers immediate, short-term, intermediate, and long-term outcomes of the DES method, which has directed the revascularization strategy in these patients facing high risk. The results of the present research will aid cardiologists in making better clinical decisions and planning bifurcation stenting procedures in the future. Therefore, the primary objective of this study is to evaluate the efficacy of different stenting techniques, such as TAP, DK-Crush, and Culotte, in the management of coronary bifurcation lesions, along with other patient features and their related immediate, short-term, and intermediate clinical outcomes.

## Materials and methods

Study design

This retrospective observational cohort study was conducted at Kulsum International Hospital, a tertiary cardiac hospital in Islamabad, Pakistan, from February 2015 to February 2021. The study aimed to evaluate the clinical outcomes of different stenting techniques in patients with coronary bifurcation lesions.

The Ethical Committee of Kulsum International Hospital, Islamabad, Pakistan issued approval 6824-89/KIH. Since the last follow-up was in December 2022, patients could be contacted directly. Therefore, no written/verbal consent was availed as the data were collected from the hospital record and not directly from the patient.

Sampling process

Patients were selected using consecutive sampling, where every patient who met the inclusion criteria during the study period was included in the study. This method ensures that all eligible patients who underwent PCI for bifurcation lesions at the hospital during the specified period were considered for inclusion, minimizing selection bias. This yielded a sample size of 172.

Inclusion and exclusion criteria

**Figure 1:**
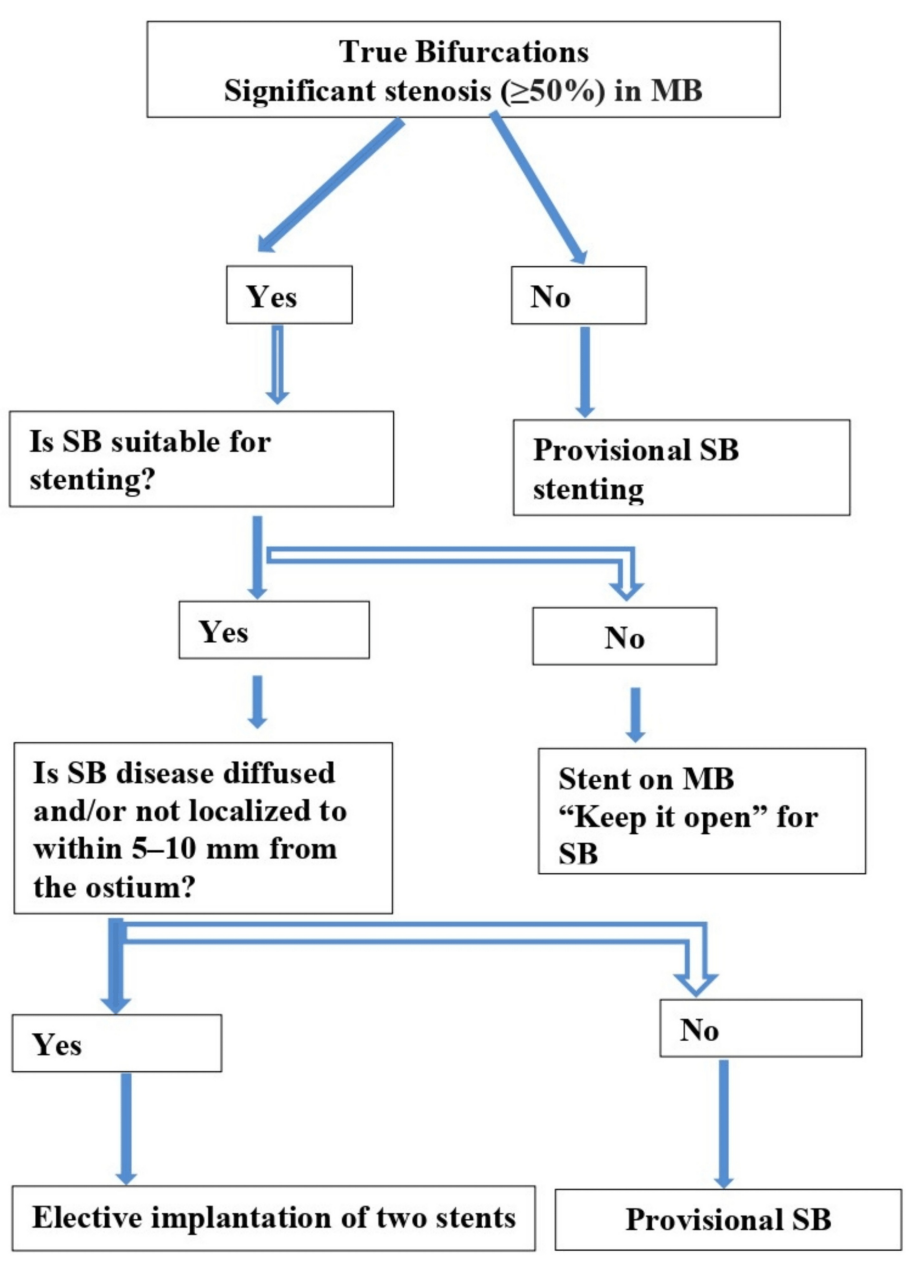
Decision theme to elective true bifurcation stenting MB: main branch, SB: side branch

Inclusion criteria were as follows: patients aged 30 to 80 years, both male/ female, diagnosed with acute coronary syndrome (ACS) or stable angina, and with or without mild renal disease (creatinine up to 2.0 mg/dl). Exclusion criteria included patients with active bleeding, significant renal impairment (creatinine > 2.0 mg/dL), contraindications to PCI, or those who had previously undergone coronary artery bypass grafting (CABG). The following scheme was adopted for bifurcation stenting (Figure 1).

Data collection methods

Data were collected retrospectively from patient registries that underwent true bifurcation stenting (two or more stents) at a tertiary cardiac center in Islamabad, Pakistan. True bifurcation lesions were assessed using the Medina criteria (1,1,1; 1,0,1; 0,1,1), with the involvement of an SB in the coronary lesion (>50% stenosis; SB of 2.5 mm or larger; lesion length of at least >5 mm mainly diffused disease; and thrombolysis in myocardial infarction (TIMI) flow of 3). The data included demographic information, clinical characteristics, procedural details, and follow-up outcomes. All data were anonymized and entered into a secure electronic database for analysis. Clinical outcomes in terms of short-term, immediate, and long-term outcomes were also documented on follow-up notes following the successful insertion of the two stents for a genuine bifurcation lesion. Immediate follow-up took place in the hospital, short-term follow-up lasted for one month, intermediate follow-up lasted for three months, and long-term follow-up took place after three years via telephone contact.

Procedural steps

The bifurcation stenting procedures were performed using various techniques, including TAP (T-stenting and small protrusion), DK-Crush, and Culotte. Each procedure followed the standard protocol: the main branch was stented first, followed by the SB, with the final kissing balloon inflation (FKBI) performed in most cases. The choice of technique was based on the cardiologist's assessment of the bifurcation anatomy and patient-specific factors.

Statistical techniques

Data were analyzed using IBM SPSS Statistics for Windows, Version 25.0 (released 2017, IBM Corp., Armonk, NY). The data analysis included the application of both descriptive and inferential statistics. Descriptive statistics, including frequencies, percentages, averages, and standard deviations, were used to summarize the data. Inferential statistics, such as the Chi-square test, were employed to examine the relationships between categorical variables. Logistic regression analysis was conducted to identify predictors of MACE. At 95% confidence intervals and 5% error margins, a p-value of <0.05 was considered statistically significant. This work is reported in line with the Strengthening The Reporting Of Cohort Studies in Surgery (STROCSS) criteria [14].

## Results

Of the 172 participants, the majority were males (69%) and only 4% were above 75 years of age. The general and clinical characteristics of the patients were analyzed, and their association with outcomes was assessed through contingency analysis. A significant relation between MACE with ACS and previous percutaneous intervention (PCI) (p < 0.000) was observed. Procedural success was good in all patients using the DES. The MAC rate was 6.9% and the FKBI, stenting technique, and bifurcation involvement were significantly associated with MACE occurrence (p < 0.01), and mortality was reported in two patients (1.16%). MACE was associated with mortality; previous PCI and hypertension increased the risk of mortality by increasing the risk of MACE.

The median age was 63 years, with the maximum being hypertensive (138; 80%). Many patients had diabetes and hypercholesterolemia (n = 72, 41.9%; n = 63, 36.6%). Approximately half of the patients who underwent revascularization procedures had previously been diagnosed with ACS (85; 49.4%). The procedural success rate was 100% with no immediate complications (within 24 hours). All patients were administered DES and were stable. Among the median classifications of bifurcation lesions, an equal percentage (n = 76; 44.2%) of patients had Medina class 0, 1, 1, and 1, 1, 1. The TAP stenting technique was used in 68 (39.5%) patients. In many patients, the SB involved in the bifurcation was the first diagonal branch (n = 66; 38%) (Table 1).

**Table 1:** Descriptive statistics of the patients; general and clinical characteristics association with major adverse cardiac events (MACE) MI = myocardial infarction, PCI = percutaneous coronary intervention, CABG = coronary artery bypass grafting, FKBI = final kissing balloon dilation, LAD = left anterior descending. Artery, D1 = first diagonal, LCX = left circumflex, OM = obtuse marginal, PDA = posterior descending artery, LMS = left main stem, PLV = posterior left ventricular, RCA = right coronary artery

Variable		Frequency	MACE (n)			P-value
			MI	TVR	No	
Age	≤63 years	87	2	2	83	0.333
	>63 years	85	6	2	77	
Gender	Male	118	6	2	110	0.672
	Female	54	2	2	50	
HTN	Yes	138	6	2	130	0.280
	No	34	2	2	30	
DM	Yes	72	2	2	68	0.586
	No	100	6	2	92	
Insulin	Yes	23	0	0	23	0.369
	No	149	8	4	137	
Previous MI	Yes	16	0	0	16	0.516
	No	156	8	4	144	
Previous PCI	Yes	35	4	4	27	0.000
	No	137	4	0	133	
Prior CABG	Yes	5	0	0	5	0.824
	No	167	8	4	155	
Acute coronary syndrome	Yes	85	8	4	73	0.001
	No	87	0	0	87	
FKBI	Yes	164	4	0	160	0.000
	No	8	4	4	0	
GP2A3B	Yes	105	4	4	97	0.226
	No	67	4	0	63	
Vascular access	RFA	116	4	4	104	0.044
	RRA	56	0	0	56	
Medina classification	0,1,1	76	2	2	72	0.049
	1,0,1	4	0	0	4	
	1,1,0	16	2	2	12	
	1,1,1	76	4	0	72	
Stent technique	TAP	68	4	0	64	0.000
	V Stenting	33	0	0	33	
	T Stenting	5	2	2	1	
	Y Stenting	8	0	0	8	
	Mini Crush	8	2	2	4	
	DK Crush	16	0	0	16	
	SKS	14	0	0	14	
	Culotte	20	0	0	20	
Bifurcation involvement	LAD+D1	66	0	0	66	
	LAD+D2	9	4	0	5	
	LAD+LCx	9	0	0	9	
	LAD+Ramus	12	0	0	12	
	LCx+OM	21	2	2	17	
	LCx+PDA	4	0	0	4	
	LAD+LMS+LCx	40	0	0	40	
	LAD+LMS+Ramus	4	0	0	4	
	PDA+PLV	3	0	0	3	
	RCA+PDA+PLV	4	2	2	0	

The TIMI flow was also measured in all patients at four intervals: initial TIMI flow in the main branch (MB), initial TIMI flow in the SB, TIMI flow in the SB after MB stenting, and final TIMI flow in the MB and SB (Table 2).

**Table 2:** Outcomes and associations (Chi-square test p-value) MB = main branch, SB = side branch, TIMI = thrombolysis in myocardial infarction, MACE = major adverse cardiac events

Variable	Death	MACE	Complications	Immediate outcomes	Short-term outcomes	Intermediate outcomes	Long-term outcomes
Initial TIMI flow in MB	0.875	0.791	0.713	0.875	0.933	0.000	0.001
Initial TIMI flow in SB	0.938	0.077	0.094	0.295	0.970	0.995	0.455
TIMI flow in SB after MB stenting	0.849	0.025	0.639	0.135	0.892	0.000	0.000
Final TIMI flow in MB	0.000	0.000	0.616	0.002	0.000	0.000	0.000
Final TIMI flow in SB	0.000	0.000	0.616	0.002	0.000	0.000	0.428
Stent technique	0.000	0.000	0.136	0.020	0.000	0.000	0.000

**Figure 2:**
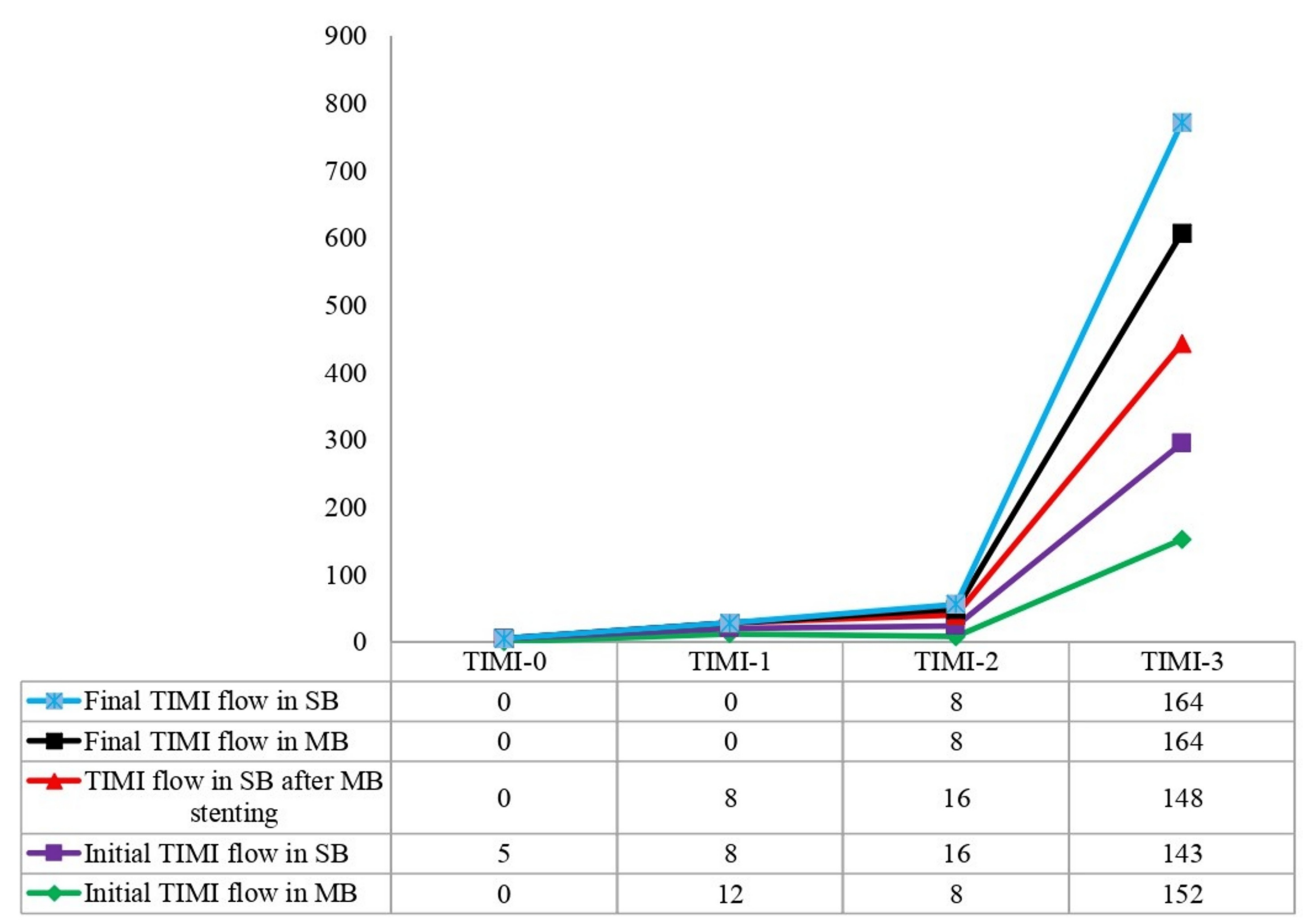
Trends in TIMI grades in stented vessels at different phases TIMI = thrombolysis in myocardial infarction, SB = side branch, MB = main branch

The final TIMI flow (MB) grades II and III were achieved in 4.6% and 88.4% of the patients, respectively (Figure 2).

**Figure 3:**
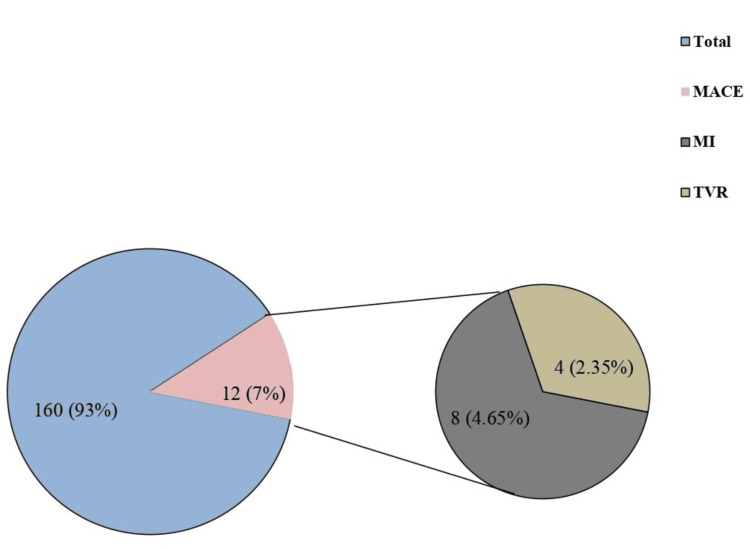
MACE distribution MACE = major adverse cardiac events, MI = myocardial infarction, TVR = target vessel

Good immediate, short-term, and long-term outcomes were observed after bifurcation stenting. The MACE rate was 12 (6.97%), among which the prevalence of MI was 8 (4.65%) and TVR was found in four patients (2.35%) (Figure 3).

**Figure 4:**
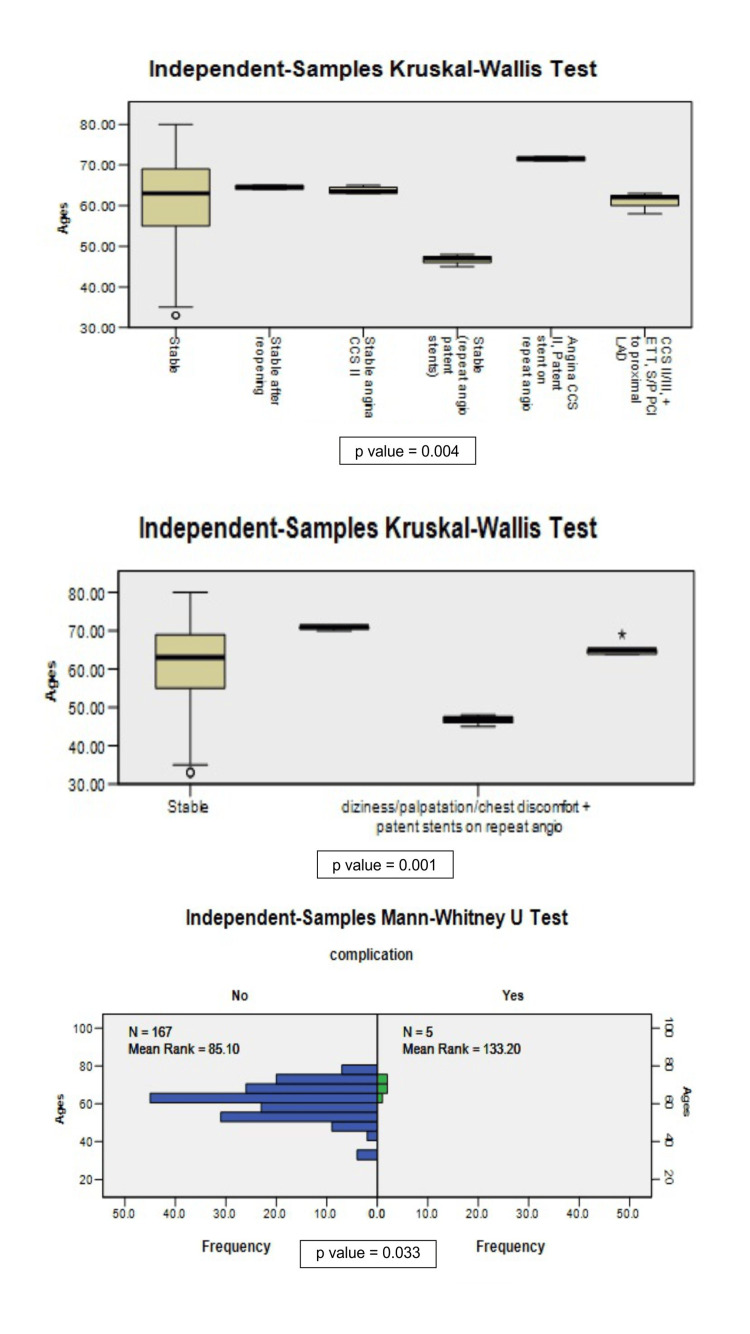
Association of age with outcomes CCS I, II, III = Canadian Cardiovascular Society grading for angina pectoris, ETT = exercise tolerance test, S/P = stable or patent, LLAD = left anterior descending artery

**Figure 5:**
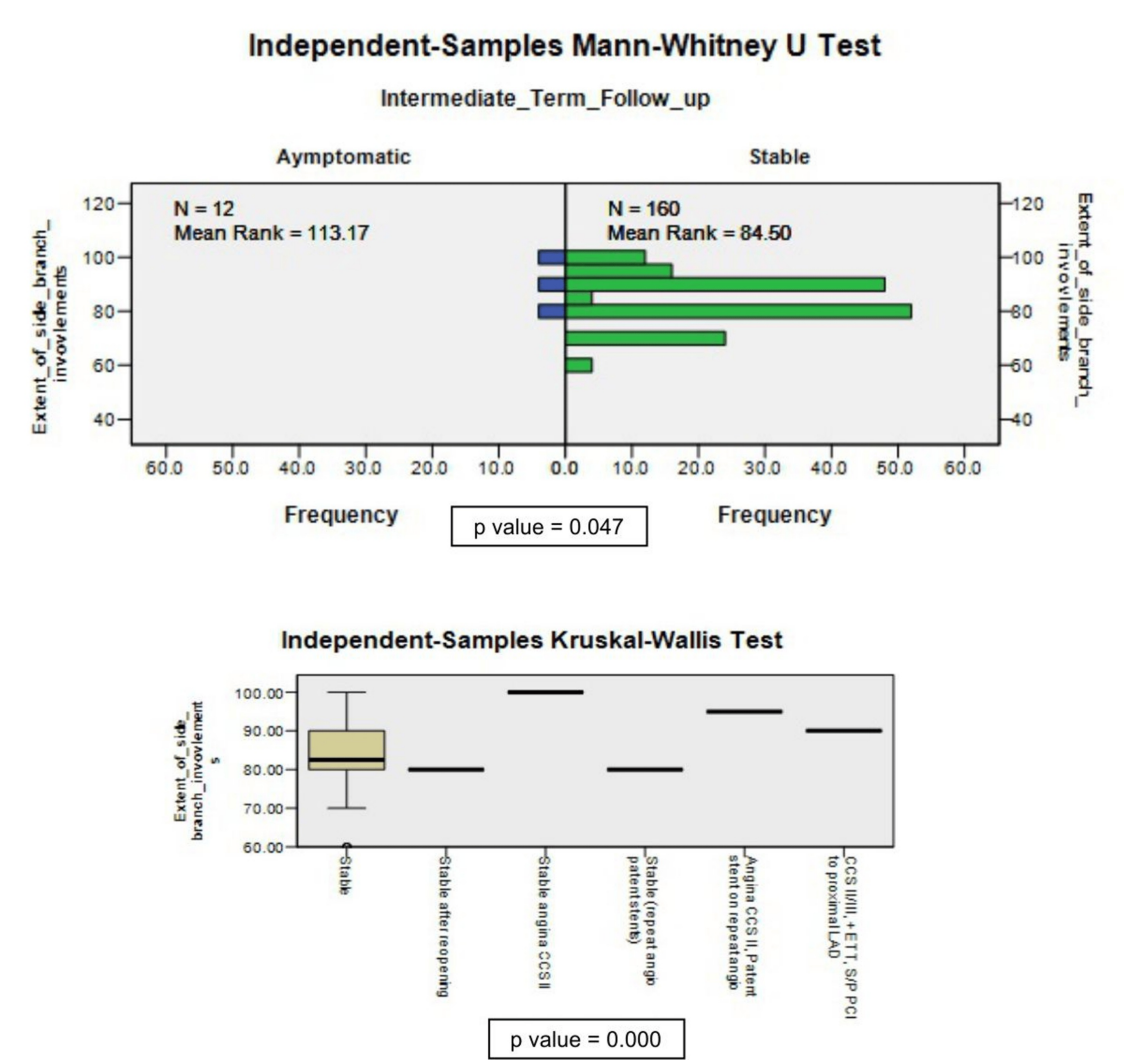
Extent of side branch (SB) involvement association with short-term and intermediate outcomes CCS I, II, III = Canadian Cardiovascular Society grading for angina pectoris, ETT = exercise tolerance test, S/P = stable or patent, LLAD = left anterior descending artery

The non-parametric tests revealed that age and the extent of SB involvement are important for outcomes (Figure 4, Figure 5).

The short-term, intermediate, and long-term outcomes were studied in detail (Table 3, Table 4, Table 5), and the factors important for them were identified.

**Table 3:** Short-term follow-up outcomes and its correlation to patient variables MI = myocardial infarction, PCI = percutaneous coronary intervention, LAD = left anterior descending artery, DI = first diagonal, LCx = left circumflex, OM = obtuse marginal, PDA = posterior descending artery, LMS = left main stem, PLV = posterior left ventricular, RCA = right coronary artery, ACS = acute coronary syndrome

Short term	Stable	CCS II-III, patent stents	Dizziness/palpitation/chest discomfort and patent stents on repeat angiography	Presented with stent thrombosis after three weeks	P-value
	159(92.4%)	4 (2.3%)	4 (2.3%)	5 (2.9%)	
Bifurcation involvement					
LAD+D1	62	0	4	0	0.000
LAD+D2	8	0	0	1	
LAD+LCx	5	4	0	0	
LAD+Ramus	12	0	0	0	
LCx+OM	17	0	0	4	
LCx+PDA	4	0	0	0	
LAD+LMS+LCx	40	0	0	0	
LAD+LMS+Ramus	4	0	0	0	
PDA+PLV	3	0	0	0	
RCA+PDA+PLV	4	0	0	0	
Diabetes mellitus					
Yes	71	0	0	1	0.064
No	88	4	4	4	
Hypertension					
Yes	133	4	0	1	0.000
No	26	0	4	4	
PCI					
Yes	31	0	0	4	0.004
No	128	4	4	1	
Hypercholestremia					
Yes	63	4	4	5	0.000
No	96	0	0	0	
Stable angina					
Yes	83	0	0	0	0.004
No	76	4	4	5	
ACS					
Yes	73	4	4	4	0.013
No	86	0	0	1	
Vascular access					
RFA	107	4	0	5	0.006
RRA	52	0	4	0	
MACE					
MI	6	0	0	2	0.000
TVR	2	0	0	2	
None	151	4	4	1	

**Table 4:** Intermediate follow-up outcomes and its correlation to patient variables MI = myocardial infarction, PCI = percutaneous coronary intervention, LAD = left anterior descending artery, DI = first diagonal, LCx = left circumflex, OM = obtuse marginal, PDA = posterior descending artery, LMS = left main stem, PLV = posterior left ventricular, RCA = right coronary artery, ACS = acute coronary syndrome

Intermediate term	Stable	Stable after reopening	Stable angina CCS2	Stable (repeat angiography patent stents)	Angina CCS II, patent stent on repeat angiography	CCS-II/III ,+ ETT,S/P PCI to proximal LAD	P-value
	152(88.4%	4 (2.3%)	4(2.3%)	4 (2.3%)	4 (2.3%)	4 (2.3%)	
Bifurcation Involvement							
LAD+D1	54	0	4	4	0	4	0.000
LAD+D2	5	0	0	0	4	0	
LAD+LCx	9	0	0	0	0	0	
LAD+Ramus	12	0	0	0	0	0	
LCx+OM	17	4	0	0	0	0	
LCx+PDA	4	0	0	0	0	0	
LAD+LMS+LCx	40	0	0	0	0	0	
LAD+LMS+Ramus	4	0	0	0	0	0	
PDA+PLV	3	0	0	0	0	0	
RCA+PDA+PLV	4	0	0	0	0	0	
DM							
Yes	71	0	0	0	0	1	0.019
No	81	4	4	4	4	3	
HTN							
Yes	129	0	4	0	4	1	0.000
No	23	4	0	4	0	3	
Previous MI							
Yes	12	0	4	0	0	0	0.000
No	140	4	0	4	4	4	
Hypercholesterolemia							
Yes	59	4	4	4	4	1	0.000
No	93	0	0	0	0	3	
Previous PCI							
Yes	31	4	0	0	0	0	0.001
No	121	0	4	4	4	4	
Smoking							
Yes	29	0	0	0	0	3	0.034
No	123	4	4	4	4	1	
Stable Angina							
Yes	79	0	4	0	0	0	0.001
No	73	4	0	4	4	4	
ACS							
Yes	73	4	0	4	4	0	0.001
No	79	0	4	0	0	4	
Access							
RFA	104	4	4	0	4	0	0.000
RRA	48	0	0	4	0	4	
Mace							
MI	2	2	0	0	4	0	0.000
TVR	2	2	0	0	0	0	
None	148	0	4	4	0	4	

**Table 5:** Long-term follow-up outcome and variables MI = myocardial infarction, PCI = percutaneous coronary intervention, LAD = left anterior descending artery, DI = first diagonal, LCx = left circumflex, OM = obtuse marginal, PDA = posterior descending artery, LMS = left main stem, PLV = posterior left ventricular, RCA = right coronary artery, ACS = acute coronary syndrome

Long-term follow-up		Symptomatic	Stable	P-value
Bifurcation involvement	LAD+D1	4	62	0.078
	LAD+D2	0	9	
	LAD+LCx	0	9	
	LAD+Ramus	0	12	
	LCx+OM	0	21	
	LCx+PDA	0	4	
	LAD+LMS+LCx	8	32	
	LAD+LMS+Ramus	0	4	
	PDA+PLV	0	3	
	RCA+PDA+PLV	0	4	
Previous MI	Yes	4	12	0.003
	No	8	148	
DM	Yes	4	68	0.53
	No	8	92	
Stable angina	Yes	4	79	0.283
	No	8	81	
ACS	Yes	8	77	0.215
	No	4	83	
Access	RFA	12	104	0.013
	RRA	0	56	
Hypercholesterolemia	Yes	12	64	0.000
	No	0	96	
MACE	MI	2	6	0.000
	TVR	0	4	
	None	0	160	
Hypertension	Yes	12	126	0.074
	No	0	34	
Previous PCI	Yes	4	31	0.246

## Discussion

The negative final outcomes of the procedure were measured as MACE, complication (stent thrombosis), and death. The positive outcomes were measured as immediate, short-term, intermediate, and long-term outcomes. Previous MI and prior CABG procedures were found to be statistically insignificant (p > 0.05) in causing MACE, while previous PCI (p = 0.000) and ACS (p = 0.001) was significantly associated with MACE. FKBI, stenting technique used, and type of vessel involved in bifurcation were also strongly associated with cardiac events (p = 0.01). MACE was also predominant in patients who underwent bifurcation stenting with minicrash stenting without FKBI. The results of this study showed that using DES for bifurcation stenting produced good procedural results (immediate term results) with a 6.97% MACE rate. The long-term, intermediate, and short-term results were largely satisfactory. The majority of the patients improved their overall health and were mostly stable. Significant correlations were observed between the occurrence of MACE and prior ACS and PCI. Most patients (88.4%) had a final TIMI flow that was also TIMI-III grade III in both arteries. TIMI-II (grade II) is associated with a higher risk of mortality as compared to TIMI-III [15], and a good percentage of patients with grade TIMI-III in our study show a good prognosis. TIMI flow is used to assess blood flow in the epicardial coronary artery. TIMI-II exhibits an impaired microcirculation [16]. TIMI flow gives an opportunity to evaluate microvascular health in the pericardial region. TIMI-III is associated with improved microvascular circulation. If the TIMI grade is less than 2, the condition is defined as microvascular obstruction (MVO) [17].

We compared TIMI flow with the outcomes of the procedure; initial TIMI flow in MB is significant for immediate and long-term outcomes. TIMI flow in the SB is significantly improved after stenting as measured in intermediate and long-term outcomes. The final TIMI flow is significantly associated with death, MACE, and immediate, short-term, intermediate, and long-term follow-up (p < 0.05) (Table 2).

As MACE predominated in procedures without FKBI and the use of the mini-rash technique, FKBI and the stenting technique had a strong connection. Our results are coherent with earlier studies that suggested the use of FKBI and DES in true bifurcation stenting with satisfactory clinical outcomes. The widespread use of DESs, which reflect a lesser hazard of clinical and angiographic restenosis, has led to satisfactory short- and long-term outcomes and technical feasibility for complex bifurcation lesions treated with PCI. In addition, new-generation DES has better associations with safety and efficacy results than first-generation DES [15,16]. Therefore, the advantages of the new-generation DES are obvious for difficult genuine bifurcation coronary lesions. Moreover, a study of the clinical results of drug-coated balloons (DCBs) indicated a strong relationship between DCB and low SB late lumen loss, although it did not indicate improved outcomes [18]. Along with notable advancements and alterations to the procedures used in PCI, the use of DES is gaining importance as a substitute revascularization strategy [19]. Similarly, there were satisfactory short-term (92.4%), intermediate (88.4%), and long-term (93%) clinical outcomes in the present study, especially in patients who underwent bifurcation in stenting with FKBI. The occurrence of MACE is directly associated with long-term outcomes. The risk of being symptomatic (in our study) at final follow-up was associated with the presence of previous MI, hypercholesterolemia, and the type of vascular access used during the procedure.

A comparative study on the use of DES in PCI and CABG procedures found a 15.4% MACE rate (including mortality, MI, and stroke). Three years of patient follow-up revealed a 14.8% MACE rate in lesions without bifurcation [20]. Comparatively, our trial, which exclusively included bifurcation coronary artery lesions, showed significantly good clinical outcomes, with a 6.97% MACE rate. This calls for attention to DES as a viable and effective revascularization approach. Another meta-analysis of nine RCTs with three-year follow-up that analyzed the long-term outcomes of true coronary artery bifurcation lesions found that the odds ratio of myocardial infarction as a MACE was 0.53 (p < 0.05), while the odds ratios for restenosis of SBs and target lesion revascularization were 1.44 and 1.59 times higher, respectively [21]. According to a comparative investigation conducted by Cho et al., 8.7% of patients who underwent left main coronary artery (LMCA) stenting experienced MACE. The clinical results for early-generation DES and current-generation DES were compared. The two-stent method and CKD were the main MACE predictors, whereas current-generation DES, CKD, and pre-intervention SB diameter stenosis of >50% were predictors [22]. By contrast, the findings of our study showed that ACS and the use of the mini-crush stent method without FKBI were also associated with MACE (MI and TLR) (p < 0.05). However, the prevalence of MACE (6.97%) agrees with the results of an earlier investigation. Another 30-year follow-up EXCEL sub-study found that TLR triggered by ischemia, cardiac mortality, MI, stroke, and the primary composite endpoint of death were common predictors of a planned two-stent strategy versus a one-stent strategy. This case was in favor of the single-stent approach. However, the DES method also yields fruitful clinical results [23]. Only the use of the DK-Crush technique has demonstrated positive clinical and long-term outcomes compared to provisional stenting in the application of the two-stent strategy [24-27]. This finding supports the findings of the current study, which show that the use of FKBI predicts positive short-term, intermediate, and long-term outcomes and that only two patients (1.16%) died.

A meta-analysis of RCTs of coronary artery bifurcation lesions reported good long-term clinical outcomes. The mortality was lower in patients who underwent the provisional stent strategy than in those who underwent the two-stenting strategy. There is no disparity in MACE between the provisional and two-stent approaches [16]. Comparative to these findings, our study results explored good outcomes with only two (2; 1.16%) mortality cases with the use of a two-stent strategy alone. The stent technique was also strongly associated with the occurrence of MACEs. However, only 6.97% of MACEs have been reported to date. Furthermore, the variations in results could be due to variations in the setup along with a very small sample size, which limits the generalization of the analysis. Angina alleviation is the ultimate objective of percutaneous intervention, so revascularization is continuously evaluated. Both the prevention of adverse cardiac events and the additional approaches were meticulously observed.

Our study's strengths include a large sample size, a long-term follow-up period of up to three years, and the comprehensive assessment of various stenting techniques in a real-world clinical setting. 

Limitations

However, the study's limitations include its retrospective nature, which may introduce selection bias, the lack of a control group or comparative analysis with non-bifurcation lesions, and the absence of routine angiographic follow-up to assess long-term stent patency.

## Conclusions

This study demonstrated that the two-stent strategy can be applied with good clinical outcomes and low complication rates in true coronary artery bifurcation lesions. The use of new-generation DES and FKBI can help reduce restenosis and minimize adverse cardiac events. The most favored technique in our study was the TAP technique, with good clinical outcomes, in addition to the universally agreed DK-Crush and Culotte techniques to treat bifurcation lesions. Potential decision-making is required regarding the use of specific stenting techniques based on the cardiologist's experience, bifurcation morphology, and RCTs. Moreover, specifically designed dedicated DES stents to vascularize bifurcation lesions with or without adjunctive therapy would be a good option where appropriate. However, further studies are required to establish the efficacy and safety of this treatment.

## References

[1] Di Gioia G, Sonck J, Colaiori I (2019). 279Clinical outcome after coronary bifurcation stenting: a systematic review and network meta-Analysis of PCI bifurcation techniques comprising 5572 patients. Eur Heart J.

[2] Louvard Y, Thomas M, Dzavik V (2008). Classification of coronary artery bifurcation lesions and treatments: time for a consensus!. Catheter Cardiovasc Interv.

[3] Collet C, Mizukami T, Grundeken MJ (2018). Contemporary techniques in percutaneous coronary intervention for bifurcation lesions. Expert Rev Cardiovasc Ther.

[4] Bogana Shanmugam V, Psaltis PJ, Tay L, Malaiapan Y, Ahmar W (2020). Procedural and clinical outcomes in management of bifurcational lesions in ST elevation myocardial infarction. Heart Lung Circ.

[5] Gwon HC (2018). Understanding the coronary bifurcation stenting. Korean Circ J.

[6] Steigen TK, Maeng M, Wiseth R (2006). Randomized study on simple versus complex stenting of coronary artery bifurcation lesions: the Nordic bifurcation study. Circulation.

[7] De Luca L (2016). Percutaneous treatment of coronary bifurcation lesions: is simplicity the ultimate sophistication?. Circ Cardiovasc Interv.

[8] Tan S, Ramzy J, Burgess S, Zaman S (2020). Percutaneous coronary intervention for coronary bifurcation lesions: latest evidence. Curr Treat Options Cardiovasc Med.

[9] Lassen JF, Holm NR, Banning A (2016). Percutaneous coronary intervention for coronary bifurcation disease: 11th consensus document from the European Bifurcation Club. EuroIntervention.

[10] Sawaya FJ, Lefèvre T, Chevalier B (2016). Contemporary approach to coronary bifurcation lesion treatment. JACC Cardiovasc Interv.

[11] Ge L, Airoldi F, Iakovou I (2005). Clinical and angiographic outcome after implantation of drug-eluting stents in bifurcation lesions with the crush stent technique: importance of final kissing balloon post-dilation. J Am Coll Cardiol.

[12] Nairooz R, Saad M, Elgendy IY (2017). Long-term outcomes of provisional stenting compared with a two-stent strategy for bifurcation lesions: a meta-analysis of randomised trials. Heart.

[13] Behan MW, Holm NR, Curzen NP (2011). Simple or complex stenting for bifurcation coronary lesions: a patient-level pooled-analysis of the Nordic Bifurcation Study and the British Bifurcation Coronary Study. Circ Cardiovasc Interv.

[14] Mathew G, Agha R, Albrecht J (2021). STROCSS 2021: strengthening the reporting of cohort, cross-sectional and case-control studies in surgery. Int J Surg.

[15] Appleby MA, Angeja BG, Dauterman K, Gibson CM (2001). Angiographic assessment of myocardial perfusion: TIMI myocardial perfusion (TMP) grading system. Heart.

[16] Doherty DJ, Sykes R, Mangion K, Berry C (2021). Predictors of microvascular reperfusion after myocardial infarction. Curr Cardiol Rep.

[17] Bangalore S, Toklu B, Amoroso N (2013). Bare metal stents, durable polymer drug eluting stents, and biodegradable polymer drug eluting stents for coronary artery disease: mixed treatment comparison meta-analysis. BMJ.

[18] Alfonso F, Fernandez C (2011). Second-generation drug-eluting stents. Moving the field forward. J Am Coll Cardiol.

[19] Megaly M, Rofael M, Saad M (2018). Outcomes with drug-coated balloons for treating the side branch of coronary bifurcation lesions. J Invasive Cardiol.

[20] Lee PH, Ahn JM, Chang M (2016). Left main coronary artery disease: secular trends in patient characteristics, treatments, and outcomes. J Am Coll Cardiol.

[21] Stone GW, Sabik JF, Serruys PW (2016). Everolimus-eluting stents or bypass surgery for left main coronary artery disease. N Engl J Med.

[22] Gao XF, Zhang YJ, Tian NL (2014). Stenting strategy for coronary artery bifurcation with drug-eluting stents: a meta-analysis of nine randomised trials and systematic review. EuroIntervention.

[23] Cho S, Kang TS, Kim JS (2018). Long-term clinical outcomes and optimal stent strategy in left main coronary bifurcation stenting. JACC Cardiovasc Interv.

[24] David K, Anthony G, Patrick S (2017). TCT-83 provisional vs. planned two-stent technique in patients with distal bifurcation left main disease undergoing PCI: the EXCEL Trial. J Am Coll Cardiol.

[25] Zhang J, Liu S, Geng T, Xu Z (2015). One-stent versus two-stent techniques for distal unprotected left main coronary artery bifurcation lesions. Int J Clin Exp Med.

[26] Chen SL, Xu B, Han YL (2015). Clinical outcome after DK crush versus Culotte stenting of distal left main bifurcation lesions: the 3-year follow-up results of the DKCRUSH-III Study. JACC Cardiovasc Interv.

[27] Chen SL, Zhang JJ, Han Y (2017). Double kissing crush versus provisional stenting for left main distal bifurcation lesions: DKCRUSH-V randomized trial. J Am Coll Cardiol.

